# Synthetic Flozins in Cancer Prevention and Combination Strategies: Structural Insights and Therapeutic Potential

**DOI:** 10.3390/molecules30244681

**Published:** 2025-12-06

**Authors:** Michał Zieliński, Olga Michalak, Magdalena Ruszczak, Marcin Cybulski

**Affiliations:** 1 Pharmacy, Cosmetic Chemistry and Biotechnology Research Group, Łukasiewicz Research Network-Industrial Chemistry Institute, Rydygiera 8, 01-793 Warsaw, Poland; michal.zielinski@ichp.lukasiewicz.gov.pl (M.Z.); olga.michalak@ichp.lukasiewicz.gov.pl (O.M.); 2 Faculty of Medicine, Medical University of Warsaw, 02-091 Warsaw, Poland; 3 Pharmaceutical Analysis Laboratory, Łukasiewicz Research Network-Industrial Chemistry Institute, Rydygiera 8, 01-793 Warsaw, Poland; magdalena.ruszczak@ichp.lukasiewicz.gov.pl

**Keywords:** (hetero)arene scaffolds, synthetic flozins, drug repurposing, cancer prevention and therapy

## Abstract

The growing number of cancer cases highlights the urgent need to develop new therapies based on effective molecules. Although anticancer activity remains a key element of oncological pharmacology, the importance of agents that support physiological functions and improve the quality of life of cancer patients is also recognised. Compounds that combine these two functions could represent a significant trend in oncology. Moreover, repurposing approved drugs for conditions beyond their original indications could be a promising strategy. An interesting example of this is synthetic flozins (SGLT2 inhibitors), which were originally designed and developed to treat type 2 diabetes mellitus. These compounds contain various aromatic and heteroaryl structures, and changes in these substituents affect the binding strength and therapeutic potency of SGLT2 inhibitors. Beyond their antidiabetic effects, SGLT2 inhibitors exert well-documented cardioprotective and nephroprotective actions, restoring metabolic homeostasis. Given these pleiotropic properties, they could benefit oncology by improving systemic functions and by directly influencing the invasion of cancer cells. This article provides an overview of the use of synthetic flozins in various contexts, with a particular focus on their potential impact on cancer biology and treatment, consolidating their position as multifunctional agents of growing systemic relevance.

## 1. Introduction

There exist several well-known examples of successful drug repurposing. Sildenafil, initially developed for coronary artery disease, was repurposed for the treatment of erectile dysfunction [1]. Thalidomide, originally an antiemetic withdrawn due to its teratogenicity, was subsequently redirected for multiple myeloma and leprosy [2]. Propranolol, commonly used in cardiology, has found its role in anxiety management [3]. Methotrexate, an anticancer agent, has been repurposed to treat autoimmune diseases [4].

**Figure 1:**
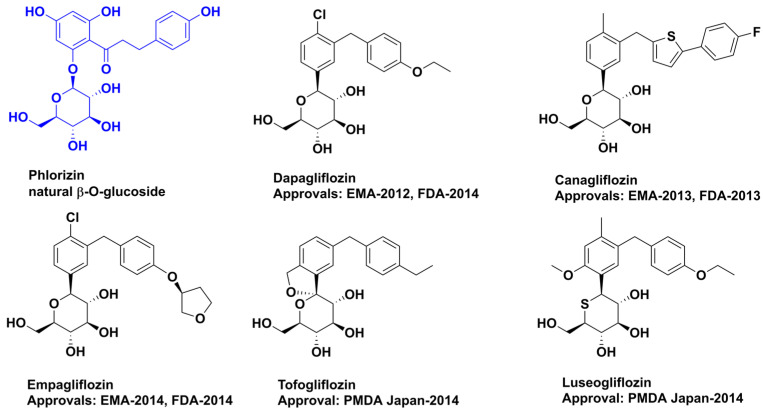
Chemical structures of phlorizin and SGLT2 inhibitors (“flozins”) discussed in this review.

Utilising existing drugs in different indications is a promising strategy, as it may provide new therapeutic alternatives for patients who have already used all well-established therapies with unsatisfactory outcomes. It also accelerates the process of drug discovery for particular diseases by relying on molecules with proven activity [5]. Sodium-glucose co-transporter 2 inhibitors (SGLT2i), also known as flozins (Figure 1), are antidiabetic drugs that have been extensively investigated in recent years for potential repurposing, with major success. As they expanded from diabetology to cardiology and nephrology, SGLT2i are a prime example of this phenomenon, with dapagliflozin and empagliflozin being incorporated into current guidelines for the treatment of chronic heart failure (HF) and chronic kidney disease (CKD). The expansion of SGLT2i indications was facilitated by the pleiotropic nature of their functions, which is well-described in the literature [6,7].

The main mechanism of action of flozins is the inhibition of the SGLT protein family, predominantly in the proximal tubules of nephrons. This blockade prevents the transport of glucose from the tubular lumen into the interstitial compartment. In patients with type 2 diabetes mellitus (T2DM), this effect, mainly mediated through SGLT2 inhibition, results in reduced renal glucose reabsorption and increased urinary glucose loss. Modulation of carbohydrate transport not only benefits diabetic patients by restoring their glucose homeostasis but could also be potentially exploited to target altered metabolism in cancer cells. Research on differences in bioenergetics between normal and neoplastic cells has a long history, with the most important discovery known as the Warburg effect. Neoplastic transformation is linked to a specific switch in catabolic cellular pathways and a shift towards glycolysis even in the presence of oxygen (aerobic glycolysis). This differs from normal cells, which, under the same conditions, rely on oxidative phosphorylation, resulting in increased glucose uptake in cancer cells compared to healthy tissues [8]. In a clinical setting, we routinely rely on this phenomenon for tumour diagnosis using PET-CT [9]. This distinct feature of neoplasms also presents a potential target for attempting to limit their ability to meet metabolic demands [10].

To avoid limitations in glucose supply, cancer cells are known to upregulate the family of facilitated diffusion glucose transporters (GLUTs). However, there is evidence that in many types of neoplasms, SGLTs are also expressed, with SGLT2, the main receptor for SGLTi, being present in pancreatic, prostatic, and lung cancer [7,11,12,13]. Inhibition of SGLT2 may hence constitute a promising, under-researched path in oncology, within which an increased number of multimorbid patients suffer from diabetes and obesity. Those two factors, which are serious problems of today’s world [14,15], have been linked to cancer promotion by several studies [16,17]. Both represent a dysregulation in glucose homeostasis, which is reported to predispose to various malignancies [18]. Therefore, drugs that can link a direct ability to suppress tumour energy supply with restoring metabolic homeostasis, and improving the functions of internal organs may constitute a beneficial trajectory in the field of oncology.

This review aims to highlight the clinical implications of various structurally distinct SGLT2 inhibitors, considering their cardioprotective, nephroprotective, and metabolic effects, as well as their potential relevance in oncology, providing a coherent perspective on their possible applications in cancer patients.

## 2. Importance of (Hetero)Arene Scaffolds in Synthetic Flozins

Aromatic rings are ubiquitous in bioactive molecules, with approximately 85% of small-molecule drugs containing a heterocyclic moiety [19]. Synthetic compounds bearing aryl and heteroaryl substituents include SGLT2 inhibitors, known as flozins. These drugs have a C-(hetero)arene-β-D-glucoside structure, in which the aromatic aglycone—usually a linked aryl or heteroaryl ring system, or a fused heterocycle—acts as a hydrophobic “anchor” within the glucose transporter’s binding pocket. The aglycones of flozins significantly influence their pharmacokinetics by affecting both affinity and selectivity for the SGLT2/SGLT1 transporter pair [20]. The origin of flozins comes from the natural compound phlorizin, an O-glucoside isolated from apple root bark and other plants (Figure 1). As has long been observed, the phlorizin induces glucosuria by blocking glucose reabsorption in the kidneys. Research on synthetic flozins began in the 1990s and led to the approval of dapagliflozin, the first drug in this class, for type-2 diabetes in 2012 (EMA 2012, FDA 2014). Subsequently, other flozins were introduced for T2DM, such as canagliflozin (FDA 2013, EMA 2013), empagliflozin (FDA 2014, EMA 2014), ertugliflozin (FDA 2017, EMA 2018) and others [21]. According to clinical trial data, including findings from DAPA-HF, DAPA-CKD, DELIVER [22], and EMPA-KIDNEY [23], the indications for SGLT2 inhibitors have been expanded to encompass HF and CKD [24]. The first expansions concerned patients with HFrEF (HF with reduced ejection fraction). Dapagliflozin, based on the DAPA-HF trial, received approvals (FDA 2020, EMA 2020, PMDA 2020), while empagliflozin, based on the EMPEROR-Reduced trial, followed shortly (FDA 2021, EMA 2021, PMDA 2021) [25]. Subsequently, the indications were extended to all patients with HF, regardless of ejection fraction. Empagliflozin was the first to secure this broad indication after the EMPEROR-Preserved trial (FDA 2022, EMA 2022, PMDA 2022), followed by dapagliflozin based on the DELIVER trial (FDA 2023, EMA 2023, PMDA 2023) [26]. The indications were further expanded to include the treatment of CKD. Dapagliflozin was the first approved for this indication based on DAPA-CKD results (FDA 2021, EMA 2021, PMDA 2021). Empagliflozin was subsequently approved based on the EMPA-KIDNEY trial (FDA 2023, EMA 2023, PMDA 2024) [27].

**Figure 2:**
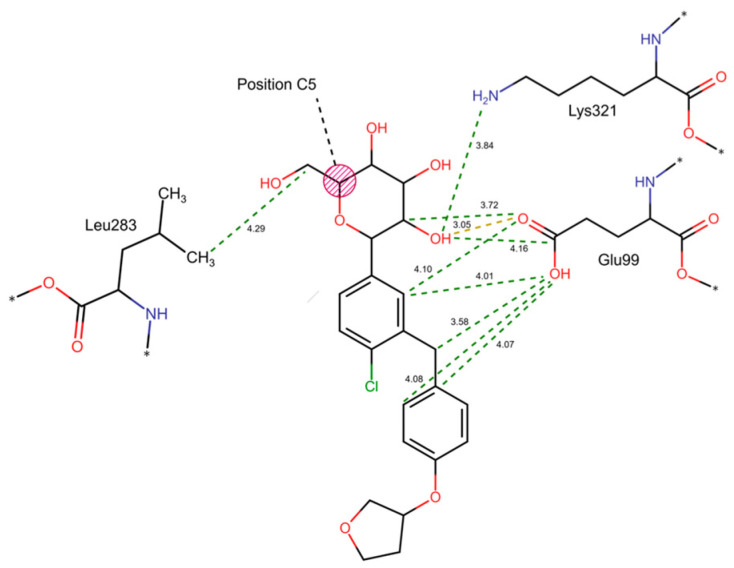
Two-dimensional representation of empagliflozin interactions with Glu99, Leu283, and Lys321; (*) indicates protein backbone attachment points. Reproduced from Molecules 2025, 30(10), 2179; Figure 9b, licenced CC BY 4.0 [29].

Multiple computational and structural studies have shown that different flozins interact differently with the SGLT2 binding pocket, and these interactions affect binding strength and inhibitory potency (Table 1). For instance, empagliflozin interacts with Ser 78, Gly 79, Lys 154, Asp 158, and Ser 393, forming stabilising hydrogen bonds and polar contacts, while its aglycone is anchored via aromatic interactions with Tyr 290 and Phe 98, as well as hydrophobic contacts with Leu 283, Val 95, and His 80 [28]. In contrast, canagliflozin engages a different set of residues—Asn 75, Ser 287, Lys 321, and Gln 457—which alters its binding geometry and slightly shifts potency and SGLT2/SGLT1 selectivity [28]. Broader analyses across many C-glycoside ligands highlight the importance of amino acid residues such as Glu 99, Leu 283, Lys 321, Asn 75, Ser 287, and Gln 457 for high-affinity binding, showing that disruption of these interactions can significantly reduce inhibitor potency [29] (Figure 2).

Molecular-dynamics simulations further support these findings, indicating that the stability of hydrophobic and polar contacts is critical for effective inhibition [30]. All SGLT inhibitors’ interactions with the target enzyme include π–π stacking with aromatic residues, van der Waals contacts with hydrophobic side chains, and local hydrogen bonds or electron-acceptor interactions involving heteroaromatic residues.

From a pharmacological perspective, the precise selection of the aglycone often increases residence time (slower dissociation), allowing for lower effective doses and convenient once-daily regimens. Moreover, these modifications are also used to optimise solubility, metabolic stability, and oral bioavailability [20,31].

## 3. General Aspects of Flozin Synthesis

**Scheme 1:**
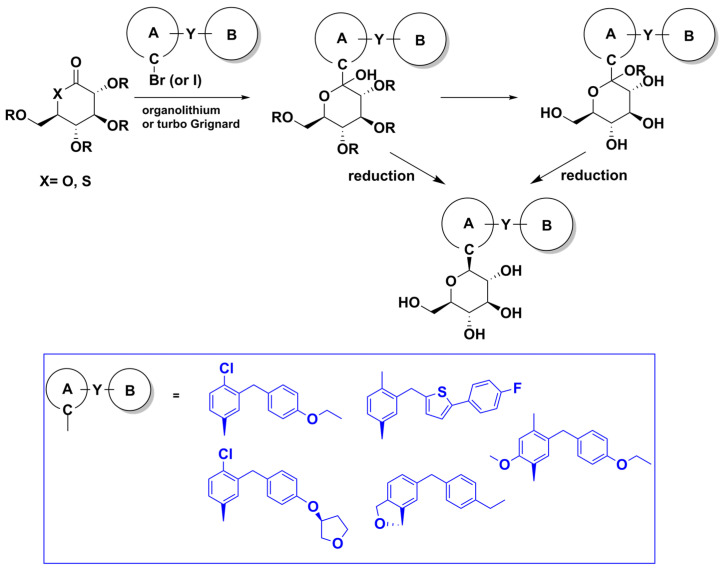
Schematic synthesis of flozins from protected D-gluconolactone.

The synthesis of flozins is based on forming a C–C bond between the sugar fragment and the aromatic aglycone, resulting in a β-C-glycoside formation (Scheme 1). It exhibits enhanced resistance to enzymatic hydrolysis, particularly intestinal enzymes, compared with natural phlorizin. Up today, glycosyl halides, gluconolactones, sugar epoxides, open-chain hemiketal derivatives, stannanes, boronate glycols, and anhydroglucose derivatives have been employed as sugar donors in flozin synthesis [32].

Among the reported approaches, the most technologically relevant are those based on protected D-gluconolactone or thio-D-gluconolactone, as in the syntheses of dapagliflozin [33], empagliflozin [34], canagliflozin [35], tofogliflozin [36,37], and luseogliflozin [38].

In these syntheses, the sugar derivative reacts with the selected aglycone in the form of an organometallic nucleophile. For dapagliflozin, empagliflozin, and canagliflozin, the in situ substitution product, bearing a TMS-protected hydroxyl group in the sugar moiety, is subsequently treated with methanol to afford a racemic mixture of C-glycosides featuring an additional methoxy group attached to the anomeric C1 carbon of the sugar ring. This intermediate is then reduced (e.g., using a trifluoroboron etherate/triethylsilane system) to yield the desired β-C-glycoside.

For luseogliflozin and tofogliflozin, condensation of the aglycone with the protected sugar affords an intermediate containing benzyl-protected hydroxyl groups, which, after reduction, leads to the formation of active SGLT inhibitors. In contrast, ipragliflozin is manufactured using activated sugar donors (such as bromoglucosides, triflate glucosides, or phosphate derivatives) that react with the appropriate aglycone in metal-catalysed C-glycosylation reactions. A representative example involves the use of arylzinc species generated by reaction with *n*-BuLi, followed by transmetalation with a ZnBr/LiBr complex under mild reaction conditions [39].

The most synthetically challenging route among those discussed is that of ertugliflozin, which proceeds through a 12-step sequence starting from tetra-O-benzyl-D-glucose. This route involves only three isolated intermediates due to the poor crystallinity of most of them. A key feature of this process is the development of a regioselective, large-scale method for the nucleophilic hydroxymethylation of ketogluconamide [40].

## 4. General Aspects of SGLT Biology

Proteins of the SGLT family, along with the GLUT proteins and the single human SWEET protein, comprise the full complement of carbohydrate transporters and sensors identified to date in the human body [41]. The former belong to the mammalian solute carrier family SLC5, which in humans comprises 12 genes encoding proteins that function as transporters or sensors, facilitating the uptake of a wide variety of solutes beyond sugars, ranging from amino acids and vitamins to organic ions [42]. SGLT proteins are sodium-driven symporters that harness the Na^+^ electrochemical gradient to transport monosaccharides such as D-glucose and D-galactose (and in some isoforms also D-mannose or D-fructose) into cells [43]. SGLT1 was the first cloned member of the family [44] and its primary role is absorption of glucose, water and sodium in the gastrointestinal tract. Studies conducted over the years on the functions of this transporter have resulted in a better understanding of treatment targeted at rehydration. Oral rehydration therapy (ORT) is now a key concept in modern medicine, directly relying on the physiological role of SGLT1. However, in contrast to SGLT2 inhibitors, the development of therapeutic agents targeting SGLT1 has not achieved major success [45].

**Figure 3:**
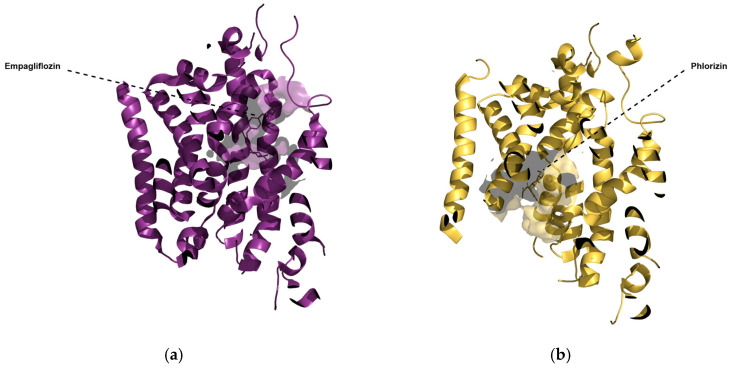
Three-dimensional representation of structures of SGLT2 with ligands bound to them. Empagliflozin (**a**) binds to the outward-open structure, whereas phlorizin (**b**) binds to the inward-open structure. Reproduced from Molecules 2025, 30(10), 2179; Figure 2, licenced CC BY 4.0 [29].

SGLT2, which shares a 60% sequence homology to SGLT1 [46], has attracted a lot of attention from medical researchers during recent years. This integral transmembrane protein, comprising 14 alpha-helices, is found in the S1 segment of the proximal convoluted tubule and predominantly mediates glucose reabsorption from the glomerular filtrate [46,47]. SGLT2 is classified as a high-capacity, low-affinity transporter, responsible for approximately 90% of glucose reabsorbed in the nephron. The physiological importance of this transporter is underscored by the fact that mutations in the gene encoding SGLT2 result in familial renal glucosuria [48]. Unlike SGLT1, its presence is restricted primarily to the kidneys, positioning it as an ideal pharmaceutical target for highly organ-specific intervention [49]. These unique characteristics have been utilised in the field of diabetology, with SGLT2 inhibitors achieving clinical prominence as a novel glucose-lowering drug group operating through a mechanism of action completely independent of insulin. Studies on conformation of SGLT2 have found differences between the binding site of naturally occurring phlorizin, which binds to the inward-open conformation, and synthetic flozins, which bind to the outward-open conformation [29] (Figure 3).

## 5. Risk of Cancer Development Under SGLT2i Treatment

Some of the most common side effects associated with SGLT2i therapy include genitourinary tract infections, euglycemic ketoacidosis and acute kidney injury [7]. However, in the context of utilising flozins in cancer therapy, it is also reasonable to discuss their potential role in inducing carcinogenesis. This is warranted when considering that another anti-diabetic agent, pioglitazone, has previously been linked to increased risk of bladder cancer [50]. The role of SGLT2i in the process of neoplastic transformation remains an undeveloped topic, and research in this area is still lacking. The occurring papers are nonuniform, presenting inconsistent results regarding the influence of this drug group on cancer risk, although more sources demonstrate no increase in cancer risk. Disproportionality analysis by Gautier et al. shows an elevated reporting odds ratio for bladder and, to a lesser extent, kidney cancer in populations taking SGLT2i [51]. It should be noted, however, that the study suggests an association between the risk of reporting bladder and kidney cancer with SGLT2i therapy but does not prove causality due to its limitations. A meta-analysis conducted by Obaid et al. has indicated a higher incidence of several malignancies, including hematologic, breast, thyroid and urinary cancers, in patients treated with SGLT2i for T2DM [52]. Nevertheless, associations in this study lacked statistical significance, and the evidence for definitive connections remains inconclusive.

On the other hand, a recent systematic review and meta-analysis compared flozins with dipeptidyl-peptidase-4 inhibitors (DPP-4i) regarding cancer incidence in people with T2DM. The study revealed that SGLT2i were associated with a significantly reduced risk of prostate, lung and liver neoplasms [53]. Early concerns that arose, particularly with the introduction of dapagliflozin, regarding an increased risk of cancer [54], have been thoroughly investigated [55,56,57]. To date, there is more literature evidence suggesting that flozins do not increase the likelihood of developing cancer than there is evidence suggesting the opposite. Current meta-analyses and systematic reviews [55,56,57] support this statement, although some conflicting results still leave room for further scientific research.

## 6. Impact on Survival in Cancer Patients

Due to the increased lifespan of people in the modern Western world and advances in therapeutic options, a clinically significant phenomenon has emerged, i.e., some cancer patients now live for many years after their initial diagnosis, often with common chronic diseases coexisting with malignancies. Chemotherapy is known to impact the functions of the cardiovascular and renal system [58,59], which are already fragile and susceptible to damage in older patients with prevalent chronic systemic conditions. This fact gave rise to novel disciplines such as cardio-oncology and nephro-oncology, which aim to view and approach patients holistically [60,61]. Considering this, SGLT2i, with their multifaceted effects, have the potential to play a pivotal role in these areas. This is especially promising, given that they have been shown to improve overall survival among patients with lung and hepatic cancers regardless of tumour characteristics or treatment type [62,63]. A retrospective cohort study on patients treated with SGLT2i in addition to chemotherapy and/or radiotherapy for gastrointestinal cancers revealed that SGLT2i use is associated with improved overall survival in male and female gastrointestinal cancer patients [64]. This association was evident for oesophageal, gastric, intestinal, anal, intrahepatic bile duct, and pancreatic cancers, but was not observed in gallbladder or extrahepatic biliary tract cancers. The authors also found that hospitalisation rates, hypoglycaemic events, acute kidney failure, and hepatic failure were lower when flozins were used in conjunction with cancer treatment, irrespective of sex. In addition to these advantages, concomitant use of SGLT2i with anticancer agents was not linked to an increased risk of adverse treatment events across any of the gastrointestinal malignancy types examined. Mao et al. studied a large cohort of individuals with T2DM and found that the addition of SGLT2i to metformin was associated with approximately 60% reduction in colorectal cancer-related mortality compared with metformin alone [65]. A recent meta-analysis including data from over 270,000 T2DM patients, published in 2025, reported promising results by linking SGLT2i use to a 30% decrease in breast cancer mortality, when compared to DPP-4i treatment [66]. Such effects of flozins on oncological outcomes are encouraging, however, further investigations in the field are still required.

## 7. Cardioprotective Effects of SGLT2i in Oncological Patients

Some members of SGLTi family revolutionised our approach to treating patients with chronic HF and are now routinely prescribed for this indication. Left ventricular myocardial dysfunction and HF are common problems in patients treated with anthracyclines, such as doxorubicin. Other cardiovascular implications linked to anticancer agents include myocarditis, mainly after immune checkpoint therapy, fluoropyrimidines-associated coronary artery disease or valvular fibrosis after radiotherapy [60]. The cardioprotective effect of flozins may hence be utilised to help mitigate these risks. A 2024 retrospective cohort analysis found that patients with a history of T2DM, cancer, and exposure to potentially cardiotoxic antineoplastic therapies benefited from the addition of SGLT2i to conventional guideline-directed medications [67]. The study looked at data from 1280 individuals and therapy outcomes over a 2-year follow-up period, showing that incorporation of SGLT2i resulted in a lower risk of acute HF exacerbation and decreased all-cause mortality. The most prevalent neoplasms included in this paper were haematological, gastrointestinal, and breast cancers. Another retrospective cohort study included a group of 933 patients aged 65 or older treated with anthracycline, and without previously diagnosed HF [68]. After analysing data from a median follow-up of 1.6 years, the authors concluded that SGLT2i exposure reduced the risk of hospitalisation due to HF. However, the data did not reach statistical significance with regard to the incidence of this disease or overall mortality. One more study involving patients with T2DM and anticancer therapy with anthracyclines revealed that SGLT2i therapy decreased the number of cardiac events in adult patients [69]. The results cited above shed light on the ability of flozins to counteract cardiotoxicity, an ever-emerging burden in oncology, and their role in improving the survival chances of cancer patients opens the possibility of a wider range of applications. As of now, three ongoing clinical trials are showing SGLT2i as an intervention in assessing their role in the prevention of cardiotoxicity (NCT06888505, NCT06844669, NCT05271162) [70]. Results from randomised clinical trials on this topic are necessary and will provide more reliable data compared to retrospective cohort studies available in medical databases.

## 8. Mechanisms of Cardioprotective Functions of SGLT2i

Cardiovascular actions of SGLT2i are well-established and commonly used in clinical settings, but the mechanisms of action behind these effects remain not completely understood. Their main feature, first utilised in their original, antidiabetic function, which is blocking SGLT2 proteins in the proximal tubules of nephrons, certainly provides benefits for the whole cardiovascular system.

However, this alone is not enough to fully account for the observable advantages of this group of drugs. Blocking renal glucose reabsorption leads to a decrease in glycaemia, which in itself is beneficial for endothelial condition and may in turn prevent micro- and macrovascular damage and complications [71], so often associated with T2DM. SGLT2i not only increase urinary glucose elimination but also promote sodium excretion through osmotic diuresis, thereby reducing the volume of the circulating plasma and blood pressure [72,73]. This basic mechanism, simple in principle, helps the cardiovascular system by reducing preload, which improves the heart’s haemodynamics. Analysing MRI data revealed that empagliflozin therapy reduces end diastolic volume, which is a favourable change in cardiac function [74]. In addition to this, SGLT2i have been reported to reduce afterload by limiting arterial stiffness and vascular resistance [75,76], which, together with lowering blood pressure, helps alleviate cardiac workload.

Heart function is also enhanced by flozins through another important mechanism. They shift myocardial and renal fuel metabolism balance from fat and glucose to ketone bodies [77]. Patients taking SGLT2i have been found to have elevated plasma levels of β-hydroxybutyrate, marking increased lipid oxidation [78]. For myocardium, one of the main organs capable of utilising these compounds, such a transition in energy source is shown to be beneficial. Ketone bodies as a fuel are more efficient, as they produce more ATP per atom of oxygen compared with glucose or free fatty acids [77]. This effect possibly plays a crucial role in supporting cardiomyocytes in the dysfunctional heart after cardiotoxic anticancer therapy, by providing them with additional energy.

**Figure 4:**
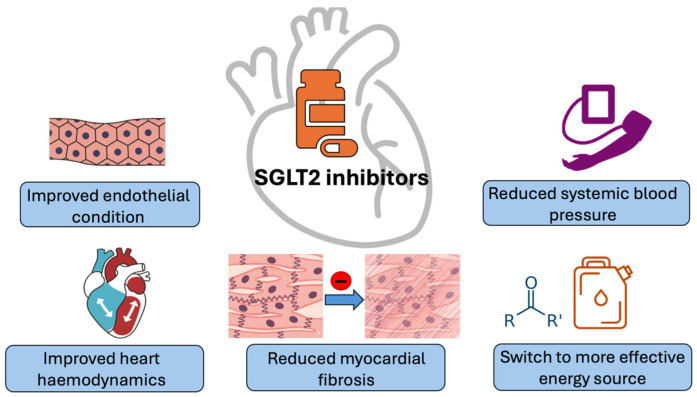
Cardioprotective mechanisms of flozins.

Insufficient heart function may be addressed by altering the disrupted structure of the myocardium. Empagliflozin has been demonstrated to alter left ventricular mass indexed to body surface [79]. The founding of reduced wall thickness is noteworthy, as greater thickness of the left ventricle is associated with the risk of sudden death [80]. Fibrosis is another element playing an important role in the pathological changes in the insufficient heart. In an animal model of myocardial infarction, dapagliflozin has been shown to halt fibrotic processes [81]. In animal models of T2DM and hypertension, the suppression of excess connective tissue accumulation by SGLT2i was also observed [82,83] (Figure 4).

## 9. Nephroprotective Effects of SGLT2i in Oncological Patients

Kidneys, like the heart, are often susceptible to damage in patients treated with chemotherapeutics. This observation has laid the groundwork for nephro-oncology, a discipline focused on kidney function in relation to anticancer therapy. It is not limited to the proper adjusting of drug doses based on altered renal clearance, but also addresses the significant issue of the direct and indirect roles of oncological drugs in the development of renal pathologies [61]. The problem is of particular importance, as approximately 20% of cancer patients may have chronic renal dysfunction [84].

Dapagliflozin has been shown to have kidney-protective effects in patients with CKD regardless of the presence of T2DM, increasing the chance of maintaining eGFR while reducing the risk of developing end-stage kidney disease and, notably, death from renal causes [85]. Empagliflozin was also studied in the same context, and the results were similar [23], thereby introducing these antidiabetic agents fully to the nephrology field. This kind of nephroprotection may play a significant role in the future of holistic oncology, as decreased eGFR and proteinuria were associated with mortality risk in patients with certain types of cancers [86]. The ability of chemotherapeutics to damage virtually every site of the nephron [87], along with the fact that immunological systemic mediators engaged in the response to cancer may impair kidney function, makes pharmacological strategies aimed at preserving glomerular capacity crucial.

## 10. Mechanisms of Nephroprotective Functions of SGLT2i

SGLT2i’s remarkable impact on the kidneys cannot be fully explained by previously mentioned reductions in blood pressure and glycaemia, especially since they are known to be rather modest diuretics and have a moderate hypoglycaemic effect compared to other drugs. Early stages of CKD may be associated with insufficient oxygen supply to the renal tissues; however, the topic of hypoxia in CKD is far more intricate than a simple decrease in oxygen delivery [88]. Nevertheless, there is a possibility that flozins improve kidney energy balance by an increase in the utilisation of non-glucose metabolic fuels, similarly to the process described in relation to the myocardium. Ketone bodies, in such a bioenergetically active organ as the kidney, may constitute a helpful role as a caloric source, since their oxidation is of greater efficiency compared to glucose [77].

Increased natriuresis, alongside glucosuria, due to SGLT2i ion channels blockade, is suspected to restore tubuloglomerular feedback through acting on the juxtaglomerular apparatus, and normalising eGFR in type 1 diabetic patients [89]. This study showed afferent arteriole vasoconstriction and a reduction in eGFR, which may seem counterintuitive in the context of the overall nephroprotective effects of flozins. Hyperfiltration is a common renal abnormality in these patients, and such a change is considered beneficial.

**Figure 5:**
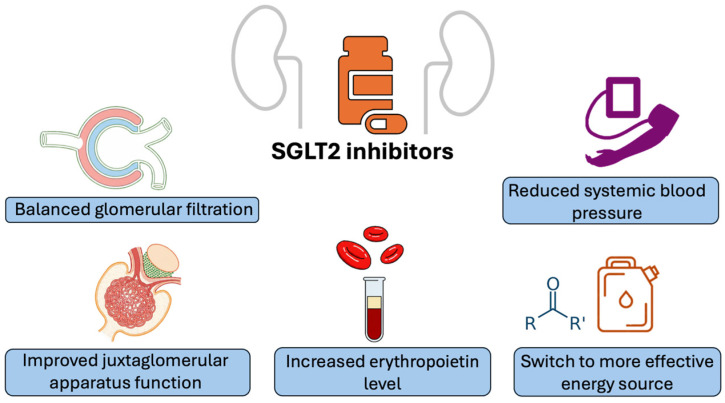
Nephroprotective mechanisms of flozins.

Through these types of alterations in local renal haemodynamics, with the addition of the putative metabolic signal of β-hydroxybutyrate, the synthesis of erythropoietin is subsequently augmented [90]. This stimulates erythrocyte production and increases haematocrit, which in turn enhances the ability of blood to transport oxygen to the tissues (Figure 5).

## 11. Impact of SGLT2i on Obesity in the Context of Cancer

Obesity and overweight have become a global problem in recent decades, even referred to as a modern-day pandemic and one of the greatest medical problems of developed countries. More than 107 million children and 603 million adults suffer from obesity, and patients with pathologically increased body weight are encountered across all areas of medicine and constitute an increasing proportion of those being treated [91]. High body mass index (BMI) is widely known to increase the risk of many diseases, with cardiovascular disease as the most well-documented and recognised [92]. As cancer emerges as one of the greatest threats to future societies, numerous studies have examined the relationship between obesity and cancer predisposition. These studies have provided findings that suggest a causation [93].

Obesity is a well-established risk for numerous cancers as well as recurrence. It is found to significantly increase the risk of developing post-menopausal breast, colorectal, endometrial, kidney, oesophageal, pancreatic, liver, and gallbladder cancer [94]. Approximately 20% of cancer cases are attributed to obesity [95]. Studies have shown that for every 5 kg/m^2^ increase in body mass index, cancer mortality increases by 10% [96]. In the case of breast cancer, obesity increases the risk of recurrence and mortality. Women who are overweight or obese at the time of diagnosis are at greater risk of poor treatment outcomes [97,98,99]. Obesity creates a specific adipose tissue microenvironment that promotes cancer progression and recurrence [100]. The mechanisms linking obesity to cancer recurrence involve multiple pathways, including insulin and insulin-like growth factors, sex steroids, adipokines, chronic inflammation, and the PI3K/Akt/mTOR signalling cascade [100]. Investigating the weight-loss effects of SGLT2i may further solidify their potential role in oncology.

A drug class effect of inducing diuretic osmosis leads to the loss of glucose in urine. This results in lowering glycaemia and reducing the availability of calories for every system of the body. Unlike another important group of T2DM drugs: glucagon-like peptide receptor agonists, they are not registered for obesity treatment, but flozins were nonetheless found to exert weight-reducing effects. A randomised, double-blind phase 3 trial demonstrated that canagliflozin at a 300 mg dose was associated with a mean weight loss of 2.9 kg in diabetic patients [101]. Another phase 3 trial with canagliflozin showed consistent results, reporting an even greater mean change (−4 kg) [102]. Interestingly, this paper noted that approximately two-thirds of the loss constituted fat tissue, while the remaining one-third was lean mass, indicating a favourable composition profile. Other SGLT2i have also been reported to decrease body weight [103,104]. Tosaki et al. examined the effect of this drug group on the fat tissue of T2DM patients and observed a significant decrease in estimated visceral fat area as well as a decrease in waist circumference [105]. Since visceral adipose tissue is known to correlate more strongly with metabolic abnormalities compared to subcutaneous deposits [106], the ability of the drug to reduce the amount of fat in this localisation represents a very favourable trait.

The observable fat-reducing effect may be interlinked with the previously described shift in metabolic energy sources in tissues under SGLT2i activity. By promoting the use of ketone bodies, these drugs accelerate lipolysis, enhance fat oxidation, and stimulate ketogenesis [78], processes that contribute to the reduction in fat reservoirs. Adipose tissue often is described as acting like an endocrine organ, being an abundant systemic source of adipokines, such as leptin. There is evidence that leptin promotes tumour cell proliferation in a variety of cancer cell types, and its elevated levels have been associated with several neoplasms, including breast, colorectal and thyroid cancers [107]. Thus, reducing fat tissue adds another rationale to the use of SGLT2i in cancer patients.

## 12. Anticancer Activity of SGLT2i

The invention of chemotherapeutics and its introduction to the medical world have helped millions of people and constituted a milestone in oncology, providing an alternative or support to often highly invasive and disfiguring surgical interventions. Unfortunately, a success over uncontrollably proliferating cells has not been achieved, as they are very prone to developing resistance to numerous agents, similarly to microbes in the field of infectious disease [108]. To address this major clinical problem, many strategies have been developed and facilitated over the years, one of them being the search for anticancer agents acting through a wide variety of mechanisms and incorporating them into polytherapy, often in expectation of a synergistic effect, or creating multiple lines of treatment. The process of developing new oncological compounds requires a lot of time and resources, so the repurposing of existing drugs, with proven safety profiles and documented antitumour activity, represents a very promising direction. In this context, the potential anticancer role of SGLT2i may be worth considering and discussing.

Preliminary trials conducted in mouse models with xenografts of pancreatic and prostate tumours revealed that canagliflozin reduced the tumour growth rate by approximately 30%, whereas dapagliflozin did not exhibit this effect. Additionally, both drugs have been reported to increase tumour necrosis by 70–100% [109]. Another study involving a xenografted hepatocellular carcinoma (HCC) model showed that canagliflozin inhibits lesion enlargement, notably independently of alterations in blood glucose levels, and significantly prolongs the survival of mice [110]. Zhou et al. subsequently evaluated the effect of dapagliflozin on breast tumour growth in a xenograft mouse model with athymic nude mice [111]. They discovered a substantial slowing of neoplasm growth and assessed tumour cell viability using Ki67 immunohistochemical staining, which confirmed the reduced proliferative capacity of these cells. Similarly, another study showed a reduction in tumour growth rate under dapagliflozin treatment in a diet-induced obesity and hyperinsulinemia mouse model of breast and colorectal cancer. The researchers also revealed that the tumour-slowing effect was insulin-dependent, and restoring high insulin levels reversed the drug’s effects [112]. Canagliflozin was found to decrease the uptake of a PET tracer reflecting glucose utilisation in early-stage lung adenocarcinomas and, when administered in the presence of premalignant lesions, significantly delayed tumour onset and markedly reduced the total tumour burden in mice [113]. In another experimental setting, canagliflozin use also led to a pronounced decrease in mean tumour weight and volume—by 35% and 56%, respectively—in a chemically induced breast cancer model in female rats [114]. The effects of canagliflozin on prostate tumour growth in mouse xenograft models using PC3 and 22RV1 cell lines were also evaluated. Mice were treated with canagliflozin administered orally in the diet, a single 5 Gy dose of radiotherapy, or a combination of both. Canagliflozin alone slowed the growth of PC3 tumours by approximately 45%, a magnitude comparable to radiation, while in 22RV1 tumours, the time to reach endpoint increased from 18 days in controls to 30 days with the drug. When canagliflozin was combined with radiotherapy, tumour progression was markedly delayed, with endpoint growth observed around 50 days. These findings were consistent across in vivo measurements and ex vivo analyses of tumour volume and weight. Kaplan–Meier survival curves further demonstrated that canagliflozin treatment improved overall survival, with the combination therapy producing the most pronounced benefit. Overall, these results highlight the significant potential of canagliflozin to slow tumour growth and enhance the effects of radiotherapy in prostate cancer models [115]. Another member of SGLT2i class, empagliflozin, was shown to regress malignant changes in the hepatic parenchyma in a mouse model of diethylnitrosamine-induced hepatocellular carcinoma. Empagliflozin treatment also reduced the serum levels of liver damage markers (AST, ALT, γ-GT) and AFP, a marker of tumour activity [116]. Additionally, high doses of this agent substantially reduced the count of lung bioluminescence as well as the number of lung nodules in a breast cancer animal model. Empagliflozin therapy was therefore suggested to lower tumour burden in the mice’s thoracic and peritoneal cavity, effectively delaying metastatic development [117]. Tofogliflozin was found to markedly reduce the number of liver tumours developing in an animal model of non-alcoholic steatohepatitis (NASH), a condition that predisposes to HCC, and increased the survival rate of treated mice [118]. It also decreased the number of colorectal pre-neoplastic lesions in diabetic and obese mice, presumably through attenuation of chronic inflammation [119].

Similarly, canagliflozin exhibited antisteatotic effects and attenuated the progression of NASH to HCC [120]. As can be seen, SGLT2i present anticancer activity in preclinical studies on a broad range of neoplasm types, which is particularly encouraging (Table 2). Animal studies, however, cannot replace human trials, and we should be cautious when extrapolating these findings to clinical settings, marking a need for further studies of high importance.

SGLT2i have the potential to augment existing therapies by exhibiting synergistic activity in combination with known anticancer agents. In an animal study, gemcitabine, a cytostatic agent representing a pyrimidine antimetabolite used in many types of cancer, was shown to reduce neoplasm growth but without inducing necrosis [12]. The authors concluded that the addition of canagliflozin to gemcitabine not only potentiated gemcitabine’s effect on pancreatic cancer tumour growth but also increased tumour necrosis. Cisplatin, a standard regimen for HCC treatment but prone to cancer resistance [123,124], has been evaluated in combination with canagliflozin in an animal model of this neoplasm. The study revealed that combining SGLT2i with cisplatin significantly decreased relative tumour volume and inhibited the development of chemotherapy resistance. Histopathological evaluation of the liver and kidney showed no evidence of distant organ damage with the addition of SGLT2i [125]. Canagliflozin has also been reported to positively contribute to the anticancer effect of sorafenib. Combining SGLT2i with this protein kinase inhibitor, which has well-established use in primary renal and liver cancer, caused a marked increase in cell death and a decrease in 3D hepatocellular cancer spheroid tumour models [126]. Although both drugs slowed xenograft tumour growth in mice, the combined treatment achieved an almost complete termination of this progression. Another chemotherapeutic whose efficacy may be enhanced by the incorporation of flozins into polytherapy is paclitaxel, an antimitotic agent inhibiting microtubule depolymerisation. In a study from 2022, dapagliflozin and paclitaxel showed a synergistic effect against breast cancer [127]. This SGLT2i member enhanced the reduction in tumour glucose uptake and improved median survival in tumour-bearing mice. The assessment of genetic drivers of breast cancer performed by the authors revealed, however, that dapagliflozin acted synergistically only in neoplasms driven by certain types of mutations and not others, emphasising the molecular intricacies of flozins’ cellular targets and pointing toward the future relevance of targeted therapies.

## 13. Molecular Mechanisms of Anticancer Activity of SGLT2i

**Figure 6:**
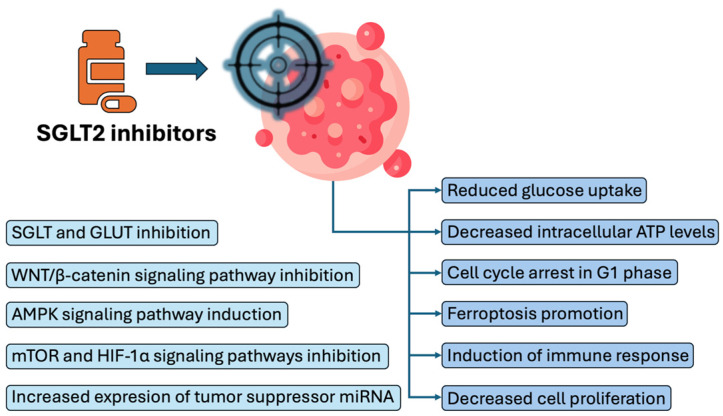
Anticancer action of flozins. AMPK: AMP-activated protein kinase; ATP: Adenosine triphosphate; GLUT: Glucose transporter; HIF-1α: Hypoxia-inducible factor 1-alpha; mTOR: Mammalian target of rapamycin; SGLT: Sodium-glucose cotransporter; SGLT2: Sodium-glucose cotransporter 2; WNT: Wingless-related integration site.

As noted earlier, SGLT2i decrease glycaemia, thus reducing systemic glucose availability for the tumour and depriving it of this preferred carbohydrate fuel. This, however, is not the only presumed mechanism of anticancer action, as flozins have much to offer in this regard, interfering with cancer cell replication, metabolism, cell cycle systems of control, and more (Figure 6).

SGLT2i not only lower systemic glucose availability but also limit its local supply to the tumour. Previously mentioned presence of SGLT2 proteins in many neoplasm types [11,12,13] offers a potential therapeutic target for restricting metabolic fuel uptake by cancer cells and thereby negatively influencing their molecular mechanisms. SGLT2i have been reported to suppress leukaemic cells from patients with adult T-cell leukaemia, by attenuation of glucose uptake, intracellular level of ATP and NADPH, which was likely linked to SGLT2 blockade and independent of apoptosis [128].

The effects of flozins on glucose transporters may not be limited to SGLT, as there is evidence that canagliflozin can nonselectively inhibit more than one isoform of GLUT [129]. In the same study, authors showed that canagliflozin attenuates glucose influx-induced β-catenin signalling activation through promoting its proteasome degradation.

Mutations of genes engaged in the WNT/β-catenin signalling pathway are significant genetic events in the development of hepatic cancers, and the overactivity of the pathway has also been implicated in cancer stemness, progression, metastasis, and drug resistance [110]. Promotion of the degradation of β-catenin by canagliflozin is a particularly promising feature, as β-catenin upregulation is tumour-specific, found in the liver tumour cells, not in the adjacent non-tumour tissues, and associated with poor differentiation [129]. Another mechanism responsible for halting cell proliferation in hepatocellular carcinoma by canagliflozin was also reported [130]. This SGLT2i drug suppressed the proliferation of Hep3B and Huh7 cells in a dose-dependent manner, and cell cycle analysis demonstrated G2/M transition arrest, with apoptosis as a secondary mechanism. In both cell populations, proteomic findings indicated that mitochondrial oxidative phosphorylation was suppressed. Consistent with that, intracellular ATP levels were decreased by canagliflozin treatment [130]. AMP-activated protein kinase (AMPK), involved in the control of metabolic energy homeostasis and cell growth, is regarded as an important tumour suppressor [131,132]. The activation of this kinase through phosphorylation was found to increase upon empagliflozin intervention [133]. Elevated phosphorylated AMPK levels were believed to lead to the inhibition of cervical cancer cell proliferation and migration, ultimately through downregulating the expression of SHH, a key regulator in the sonic hedgehog pathway. Aberrations in this pathway have been linked to the development of diverse neoplasms in humans and to the phenomenon of drug resistance [134]. Empagliflozin has further been reported to increase the expression of miR-128-3p, a tumour suppressor miRNA often downregulated in breast cancer. This change was associated with reduced expression of CD98hc, which in turn predisposes breast cancer cells to ferroptosis, a form of programmed cell death dependent on iron metabolism dysregulation [117]. In prostate cancer studies, canagliflozin showed inhibition of the activity of mitochondrial complex I, leading to the activation of AMPK and followed by the inhibition of mechanistic target of rapamycin complex 1 (mTORC1) and hypoxia-inducible factor 1-alpha (HIF-1α) pathways. This resulted in reduced cell proliferation and survival. Canagliflozin also induced transcriptional reprogramming by inhibiting the expression of genes associated with poor prognosis in prostate cancer, including genes linked to the MAPK/ERK pathway. These effects were observed in both androgen-sensitive and androgen-insensitive prostate cancer models [115]. SGLT2i exhibit multifaceted actions at the cellular level through multiple molecular mechanisms, resulting in a broad range of biological outcomes (Table 3).

## 14. Materials and Methods

Two independent researchers searched the medical database PubMed and ASC Publications database using phrases including the name of the drug group or its specific members and additional terms. Phrases used in the search included “SGLT2i synthesis”, “SGLT2i cancer”, “SGLT2i nephroprotection”, “SGLT2i cardioprotection mechanisms”, “canagliflozin anticancer”, etc. We included original papers, systematic reviews, narrative reviews, and meta-analyses published in English. References from identified papers were screened, and additional relevant studies were incorporated where appropriate. In total, we collected 152 articles, of which 137 were ultimately selected, including papers discussing epidemiological aspects and cancer-related topics. Studies briefly mentioning SGLT2i in the context of cancer were excluded. The graphical abstract and Figure 4, Figure 5 and Figure 6 were generated using Gemini 2.5 Pro based on original Figures created by the authors. No generative AI tools were used to create or modify the scientific data or textual content.

## 15. Conclusions

SGLT2is represent a promising group of drugs with considerable potential to enter oncology and provide additional therapeutic value for many patients. Specific modifications within the flozin scaffold, especially in the aromatic fragments, appear to be a crucial, yet underexplored, avenue for possibly enhancing the anticancer effect. This points to a significant R&D opportunity for the design of novel, next-generation SGLT2i, which would exhibit a potent tumour growth inhibition. Engineering bifunctional agents that could concurrently target oncogenic pathways and restore altered metabolic homeostasis constitutes a fertile ground for medicinal and synthetic chemists. In order to progress in this area, research on complex relationships between structure and activity is needed.

Based on the reviewed literature, flozins appear safe with respect to the risk of carcinogenesis and have been shown in numerous studies to exhibit a positive effect on the course of cancer. Longer-living cancer patients increasingly require long-term, comprehensive internal medicine care and a holistic approach, with flozins fitting very well into such a vision of medical oncology. Their profound positive effects on the interconnected cardiovascular and renal systems may translate into improved patient outcomes and enhanced quality of life. In addition to their systemic activity, SGLT2i can act on a plethora of mechanisms directly affecting tumour cells. The identified anticancer actions included the ability to suppress malignant proliferation, and it is worth noting the potential of flozins to enhance the activity of widely used chemotherapeutics such as gemcitabine or sorafenib. The observed synergism reinforces the notion that SGLT2i may have a place in the oncology of the future. Taken together, the pleiotropic systemic effects of SGLT2 inhibitors—spanning cardiovascular, renal, metabolic and inflammatory domains—create a strong rationale for exploring their role in oncology, both as supportive therapy and as potential direct anticancer agents.

## Figures and Tables

**Table 1:** The selected interaction of flozins with SGLT2.

Flozin	Hydrogen-Bond/Polar Residues	Aromatic Residues	Hydrophobic Residues	Reference
Empagliflozin	Ser 78, Gly 79, Lys 154, Asp 158, Ser 393	Tyr 290, Phe 98	Leu 283, Val 95, His 80	[28]
Dapagliflozin	Arg 46, Arg 49, Ser 78, Ser 393	not specified	Ile 76, Met 216	[28]
Canagliflozin	Asn 75, Ser 287, Lys 321, Gln 457	Tyr 150, Tyr 290, Trp 289	Val 95, His 80	[28]
General C-glycosides	Glu 99, Leu 283, Lys 321, Asn 75, Ser 287, Gln 457	Phe 98, Tyr 290, Trp 291	Leu 84, Val 95	[29]

**Table 2:** Preclinical anticancer studies of SGLT2i on animal models.

Studies	SGLT2i Member	Cancer Type	Findings
Wright et al. (2020) [109]	Canagliflozin and Dapagliflozin	Pancreatic cancer, Prostate cancer	Canagliflozin ↓ tumour growth rate ~30%; dapagliflozin no effect; ↑ tumour necrosis 70–100% (both)
Hung et al. (2019) [110]	Canagliflozin	Hepatocellular carcinoma (HCC)	Inhibited lesion enlargement; prolonged survival; effect independent of blood glucose changes.
Zhou et al. (2020) [111]	Dapagliflozin	Breast cancer	Slowed tumour growth; Ki67 staining confirmed ↓ proliferative capacity of tumour cells.
Nasiri et al. (2019) [112]	Dapagliflozin	Breast cancer, Colorectal cancer	Reduced tumour growth rate; insulin-dependent effect
Scafoglio et al. (2018) [113]	Canagliflozin	Lung cancer	↓ PET tracer uptake (glucose utilisation), delayed tumour onset, and markedly reduced total tumour burden.
Sabaa et al. (2022) [114]	Canagliflozin	Breast cancer	↓ tumour weight and volume
Ali et al. (2023) [115]	Canagliflozin	Prostate cancer	Slowed tumour growth, without toxicity; sensitisation to radiotherapy
Abdelhamid et al. (2022) [116]	Empagliflozin	HCC	Regression of malignant changes; ↓ AST/ALT/γ-GT and AFP (tumour activity marker).
Nalla et al. (2024) [117]	Empagliflozin	Breast cancer	↓ lung bioluminescence and the number of lung nodules; lowered thoracic/peritoneal tumour burden; delayed metastatic development.
Murakami et al. (2022) [118]	Tofogliflozin	HCC	Markedly ↓ the number of liver tumours and ↑ the survival of treated mice.
Kato et al. (2019) [119]	Tofogliflozin	Colorectal cancer	↓ number of pre-neoplastic lesions, presumably via attenuation of chronic inflammation.
Shiba et al. (2018) [120]	Canagliflozin	HCC	Antisteatotic effects; attenuation of progression from NASH to HCC.
Fayed et al. (2025) [121]	Canagliflozin	HCC	↓ tumour progression markers: AFP, AFU and CEA, levels; ↓ oxidative stress marker: MDA levels; ↑ antioxidant markers: SOD and GSH levels.
Ding et al. (2023) [122]	Canagliflozin	Lung cancer	↓ tumour growth by engaging the immune system and decreasing tumour immunosuppressive activity; ↑ inflammatory response in the tumour microenvironment

Abbreviations: SGLT—sodium–glucose cotransporter; HCC—hepatocellular carcinoma; AFP—alpha-fetoprotein; AFU—alpha-L-fucosidase; CEA—carcinoembryonic antigen; AST—aspartate aminotransferase; ALT—alanine aminotransferase; γ-GT—gamma-glutamyl transferase; NASH—non-alcoholic steatohepatitis; SOD—superoxide dismutase; GSH—glutathione.

**Table 3:** Molecular mechanisms of SGLT2i.

Studies	SGLT2i Member	Cancer Type	Mechanism	Biological Effect
Nakachi et al. (2025) [128]	Luseogliflozin and Tofogliflozin	Leukaemia	Glucose intake inhibition through SGLT2 blockade	Suppression of the proliferation and colony formation of cells
Hung et al. (2019) [110]	Canagliflozin	HCC	Nonselective inhibition of >1 GLUT isoform; ↓ glucose influx → PP2A inhibition → proteasomal degradation of β-catenin; attenuation of WNT/β-catenin signalling	Reduced proliferation and colony formation, slowed xenograft tumour growth
Nakano et al. (2020) [130]	Canagliflozin	HCC	Suppression of mitochondrial oxidative phosphorylation (OXPHOS); ↓ intracellular ATP	G2/M arrest; secondary apoptosis; ↓ proliferation
Xie et al. (2020) [133]	Empagliflozin	Cervical cancer	AMPK activation (↑ phosphorylation) → downregulation of SHH	↓ proliferation and migration
Nalla et al. (2024) [117]	Empagliflozin	Breast cancer	miR-128-3p ↑ → CD98hc ↓ → sensitization to ferroptosis	↓ cell viability, ↑ ferroptotic markers (lipid ROS)
Ali et al. (2023) [115]	Canagliflozin	Prostate cancer	Inhibition of mitochondrial complex I → AMPK activation → inhibition of mTORC1 and HIF-1α; transcriptional reprogramming (↓ poor-prognosis genes, MAPK/ERK)	↓ proliferation and survival
Okada et al. (2020) [135]	Dapagliflozin	Colorectal cancer	↑ ADAM10 activity → proteolytic cleavage/shedding of DDR1 ectodomain → ↓ DDR1 signalling → loss of adhesion to collagen I/IV	Detachment and loss of adhesion; reduced cell viability
Wu et al. (2022) [136]	Canagliflozin	Osteosarcoma	Inhibition of AKT phosphorylation → activation of STING/IRF3/IFN-β innate immunity pathway; induction of immune infiltration	↓ tumour growth; ↑ infiltration of CD4^+^ and CD8^+^ T cells; activation of innate immune response; synergistic antitumour effect with STING agonist
Kuang et al. (2017) [137]	Dapagliflozin	Renal cell carcinoma	↓ glucose uptake; downregulation of SGLT2; alteration in cell cycle	↓ proliferation and viability; cell cycle arrest (G1), increased apoptosis

Abbreviations: HCC—hepatocellular carcinoma; SGLT2 –sodium-glucose cotransporter 2; GLUT—glucose transporters; PP2A—protein phosphatase 2A; OXPHOS—oxidative phosphorylation; ATP—adenosine triphosphate; AMPK—AMP-activated protein kinase; SHH—sonic hedgehog; miR-128-3p—microRNA-128-3p; CD98hc—cluster of differentiation 98 heavy chain; mTORC1—mechanistic target of rapamycin complex 1; HIF-1α—hypoxia-inducible factor 1-alpha; MAPK—mitogen-activated protein kinase; ERK—extracellular signal-regulated kinase; ADAM10—a disintegrin and metalloproteinase domain-containing protein 10; DDR1—discoidin domain receptor 1; AKT—protein kinase B; STING—stimulator of interferon genes; IRF3—interferon regulatory factor 3; IFN-β—interferon beta; WNT/β-catenin—Wingless-integrated/β-catenin; ROS—reactive oxygen species; CD4^+^ and CD8^+^ T cells—cluster of differentiation 4 and 8 positive T cells.

## Data Availability

This study did not generate any new data. All data supporting the findings of this study are available within the article.

## Abbreviations

The following abbreviations are used in this manuscript:

ADAM10	a disintegrin and metalloproteinase domain-containing protein 10
AFU	alpha-L-fucosidase
AFP	alpha-fetoprotein
AKT	protein kinase B
AMPK	AMP-activated protein kinase
ALT	alanine aminotransferase
AST	aspartate aminotransferase
ATP	adenosine triphosphate
CD4+ T cells	cluster of differentiation 4-positive T cells
CD8+ T cells	cluster of differentiation 8-positive T cells
CD98hc	cluster of differentiation 98 heavy chain
CEA	carcinoembryonic antigen
CKD	chronic kidney disease
DPP-4i	dipeptidyl peptidase-4 inhibitors
DDR1	discoidin domain receptor 1
ERK	extracellular signal-regulated kinase
GSH	glutathione
GLUT	glucose transporters
γ-GT	gamma-glutamyl transferase
HCC	hepatocellular carcinoma
HFrEF	heart failure with reduced ejection fraction
HF	heart failure
HIF-1α	hypoxia-inducible factor 1-alpha
IFN-β	interferon-beta
IRF3	interferon regulatory factor 3
MAPK	mitogen-activated protein kinase
miR-128-3p	microRNA-128-3p
mTORC1	mechanistic target of rapamycin complex 1
NASH	non-alcoholic steatohepatitis
OXPHOS	oxidative phosphorylation
PP2A	protein phosphatase 2A
ROS	reactive oxygen species
SGLT	sodium–glucose cotransporter
SGLT2	sodium–glucose cotransporter 2
SHH	sonic hedgehog
SOD	superoxide dismutase
STING	stimulator of interferon genes
WNT/β-catenin	wingless-integrated/β-catenin
